# Food prices and food crises since 2020: evidence from Mali, northeast Nigeria, Sudan, and northern Uganda

**DOI:** 10.1111/disa.70037

**Published:** 2026-01-21

**Authors:** Steve Wiggins, Bilkisu Yayaji Ahmed, Betty Akullo, Boukary Barry, Johnson Dudu, Job Eronmhonsele, Yusuf Kiwala, Dicta Ogisi, Andrew Onokerhoraye, Jimmy Opio, Neema Patel, Hussein Sulieman

**Affiliations:** ^1^ ODI Global United Kingdom; ^2^ Centre for Population and Environmental Development Nigeria; ^3^ Women and Rural Development Network Uganda; ^4^ Kéné Conseils Mali; ^5^ Independent Consultant United Kingdom; ^6^ Centre for Remote Sensing and GIS University of Gadarif Sudan

**Keywords:** commodity prices, coping, food security, Mali, Nigeria, Sudan, Uganda

## Abstract

When world prices of maize and wheat doubled between early 2020 and mid‐2022, it was feared the increases would transmit to markets in the Global South, threatening the food security of vulnerable people. We report studies conducted in Mali, northeast Nigeria, Sudan, and northern Uganda to examine changes in the prices of cereals, their consequences, and public responses. From early 2020, the prices of staples in the four countries rose strongly, doubling or more, and remained high up to the time of writing (mid‐2025). Price increases resulted largely from domestic factors, above all failed harvests and, in Mali and Sudan, conflict: world prices played only a minor role. People on low incomes economised on food, cut spending on health and education, and tried to cope by finding extra work, selling off assets, and borrowing money—but not always successfully. Public support has been scant: most people have had to manage using the resources of family, neighbours, and local communities.

## 1 BACKGROUND: WORLD PRICES AND FOOD CRISES

### 1.1 World prices

**FIGURE 1:**
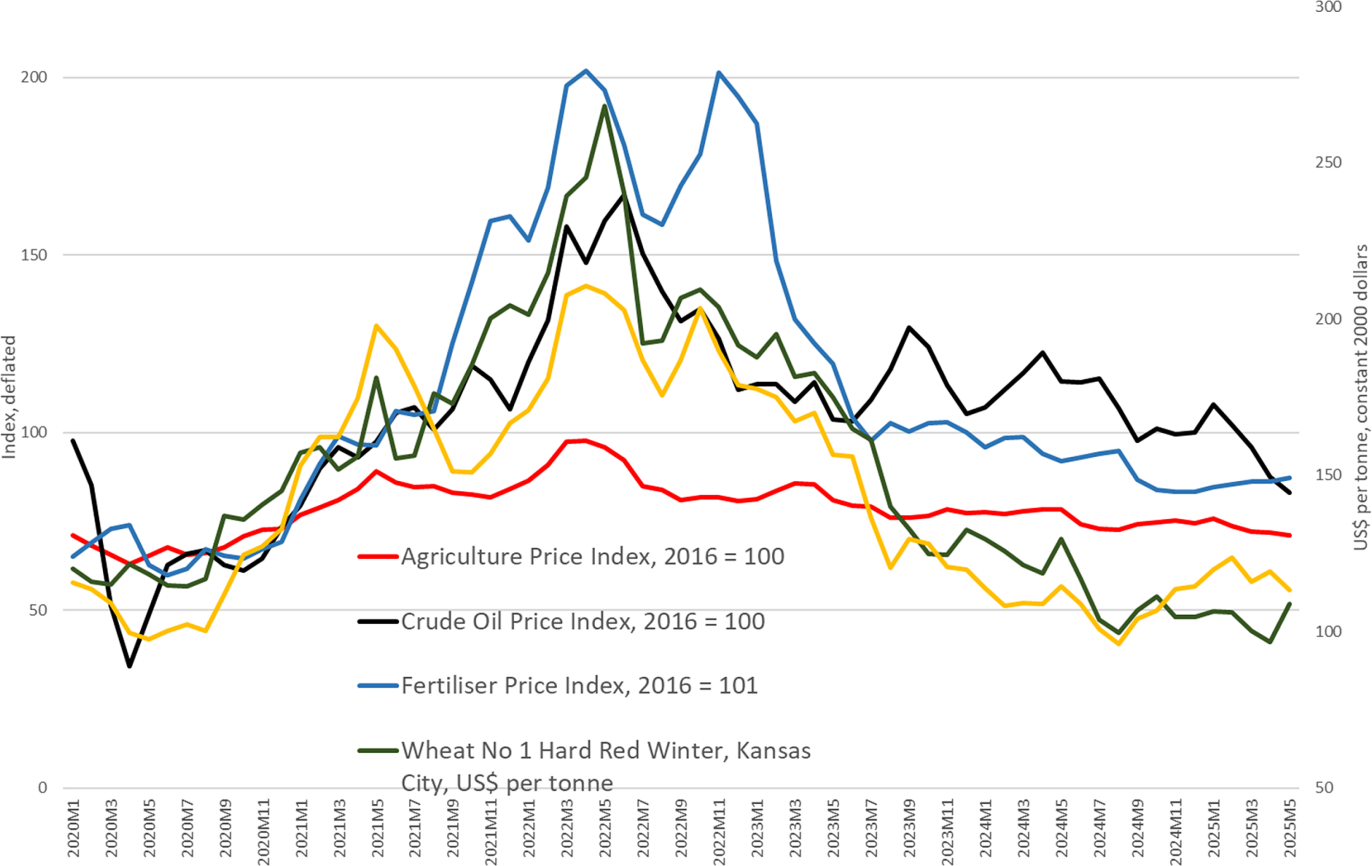
The 2022 spike in fertiliser, food, and oil prices on world markets: commodity prices from January 2020–May 2025. **Source:** authors, compiled using International Monetary Fund primary commodity prices, with indices and prices deflated using the United States gross domestic product deflator, based on July 2000.

From the first quarter of 2020, world prices of cereals, fertiliser, oil, and other commodities began to increase, peaking in May–June 2022 (see Figure 1). More specifically, during that time, prices, in constant terms, rose by 181 per cent for crude oil, by 144 per cent for (an index of) fertiliser, by 28 per cent for the agriculture index, by 73 per cent for maize, and by 111 per cent for wheat. The upsurge in world prices was compounded by Russia's invasion of Ukraine in late February 2022.

The shock of the invasion led to concerns about the loss of exports of cereals and fertiliser from Ukraine, and possibly also from Russia and Kazakhstan, both significant grain exporters shipping from Black Sea ports. Researchers at universities, think‐tanks, and aid agencies and journalists in the business press sounded the alarm regarding the possible consequences for people living on low incomes in the Global South, above all those in countries which imported a substantial amount of the domestic supply of cereals.^1^ At worst, commentators argued, if Ukraine's exports were lost entirely (and supplies from Russia and Kazakhstan were also reduced), countries in the Global South might be unable to import the quantity of cereals they usually did. At best, the prices of those imports would be elevated significantly, driving up prices in domestic markets: people on low incomes would see alarming increases in the prices of staple foods. Dire warnings were made about vulnerable people being unable to afford to eat sufficiently, with consequent malnutrition.

In the years since the invasion, world prices have fallen back; by May 2025, the prices of maize and wheat, and the agriculture price index, had returned to their levels of early 2020. Fertiliser and oil prices also decreased but remained a little above their levels of early 2020. In the almost six years since January 2020, it seems that commodity prices have completed a cycle: something quite frequently seen with commodity prices.^2^

These concerns led, in 2022, the United Kingdom's Foreign, Commonwealth & Development Office (FCDO) to commission researchers as part of an FCDO‐funded consortium, Supporting Pastoralism and Agriculture in Recurrent & Protracted Crises (SPARC). The objective was to explore the potential impacts of higher world prices within the countries covered by the consortium: the Sahel from Mauritania to Somalia, plus Syria and Yemen. Rapid early assessments were conducted, drawing on existing data on food systems in potentially vulnerable countries, to think through the implications of higher prices.^3^ Early assessments were followed by a closer examination of what had happened to domestic food prices in Mali and Sudan since 2000, with what effects, and with what public responses. The two countries were chosen owing to contrasts in their food systems: Mali growing almost all cereals consumed; and Sudan being heavily dependent on wheat imports to bake bread, the urban staple. Higher international prices should barely have affected landlocked Mali, but the consequences for Sudan were expected to be severe.

A short technical report (Wiggins et al., 2023) soon showed that the domestic price of cereals had doubled in Mali and gone up by 10 or more times in Sudan since 2020; but these increases could be attributed in very large part to domestic forces rather than the movement of international prices. Such large upsurges in prices meant that people on low incomes were facing a food crisis.

Subsequently, in 2023, Canada's International Development Research Centre commissioned, in partnership with SPARC, studies of food price increases in northeast Nigeria and northern Uganda. These analyses would reveal large increases in food prices from 2020, also with domestic causes at the fore. Both regions confronted food crises; perhaps similar to those that have periodically arisen in Africa's history.

### 1.2 Food crises in Africa

According to the International Food Policy Research Institute, food crises are:

*shocks to food systems that lead to severe disruptions and cause a surge in acute food insecurity. Food system shocks have diverse causes and impacts*.^4^

Such shocks run on a spectrum from food crisis to food emergency to famine: distinctions formally defined in the Integrated Food Security Phase Classification (IPC) used by humanitarian relief agencies.^5^

**FIGURE 2:**
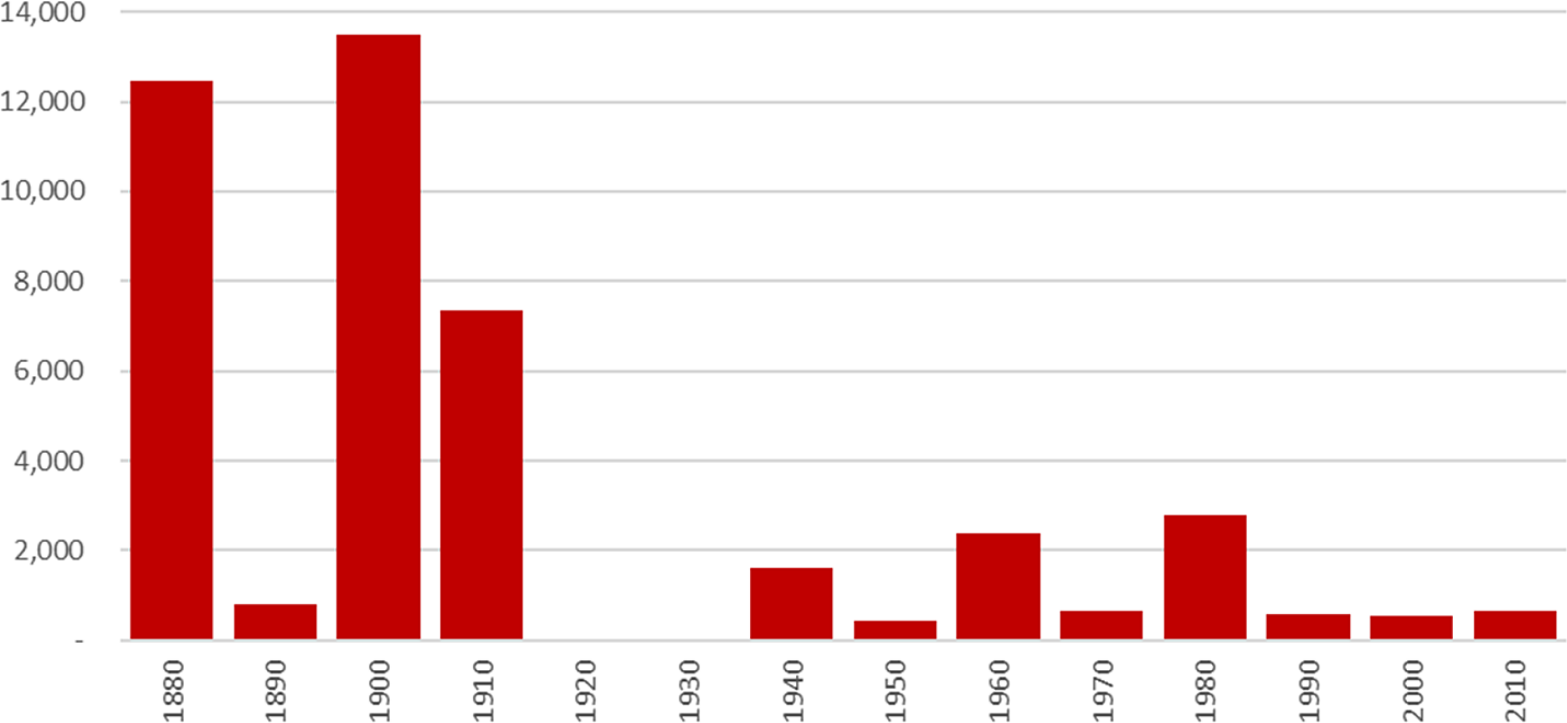
Estimated mortality to famine in Africa, persons dying per million, by decade from the 1880s–2010s. **Source:** authors, compiled from famines listed on the website of the World Peace Foundation^7^, plus the Rwandan famine of 1943–44 (Devereux, 2000), as compared to population estimates: 1880s–1940s from the Frankema‐Jerven African Population Database 1850–1960, version 3.0^8^; and 1950s onwards from FAOSTAT of the Food and Agriculture Organization of the United Nations^9^.

That the incidence of food crises, mainly in Africa, but also in another 17 vulnerable countries, has risen from 2016 to (at least) 2023 (FSIN and Global Network Against Food Crises, 2024), may obscure their longstanding record, including of outright famine, in Africa. At least 33 famines have been recorded on the continent since 1880. Comparing estimated mortality to those famines for each decade with the estimated population of Africa at mid‐decade, mortality to famine has fallen markedly since the 1980s from the levels seen before, above all the very high rates from the 1880s to the 1910s when European powers invaded Africa (see Figure 2). The increased occurrence of food crises since 2016 is a cause for concern, but a longer perspective suggests that such trends are far from^6^ unprecedented.

Three features of food crises, and especially famines, stand out from the historical record. First, the causes of crises often include violence, such as war, civil war, punitive massacres, pillage, forced displacement, and ethnic cleansing. Of the 33 instances of famine since 1880, no fewer than 24 include violence as a substantial cause of famine. Natural hazards, above all drought and livestock disease, are the other major cause.

Second, famines are not just times when food runs scarce, but also times when staple food prices rise by multiples of previous prices. For example, when famine struck Darfur, Sudan, in 1984–85, the price of sorghum increased approximately five times between mid‐1983 and mid‐1985 (see Figure 5.3 in de Waal, 1989). The effect of such price rises for anyone who had to buy food—many smallholders may grow enough to cover their requirement of staples for most of the year, but often they must buy in extra food during the lean season prior to the next harvest—was huge: they variously ate less and switched to the cheapest possible alternatives, which included foraging for wild foods. As Nobel Prize winner Amartya Sen (1981) argued, the problem during famines is not so much that food availability declines, but that the entitlement of people on low incomes to food falls precipitously.

Third, famines are not only about starvation, they are also times of disease—frequently, by far the largest cause of mortality during famines (de Waal, 1989; Dyson, 1993; Swift, 1993)—and times when vulnerable people lose vital assets—livestock, tools, seeds, and land—and become destitute.

## 2 QUESTIONS, METHODS, AND DATA

This paper brings together the findings of three sets of studies of higher food prices in four countries: Mali, Sudan (Wiggins et al., 2023; Wiggins et al.,  forthcoming), northeast Nigeria (Onokerhoraye et al., 2025), and northern Uganda (Akullo et al., 2025). Each study posed three common questions (among others):

1. What has happened to the prices of staple foods since 2020? What explains food price movements?
2. What have been the impacts of price changes on people on low incomes? What spending and consumption have they had to cut? How have they looked for additional income?
3. What responses to higher food prices and their impacts were forthcoming from public agencies—government, civil society organisations (CSOs), non‐governmental organisations (NGOs), and donors? How effective have those been?

The studies of prices in Mali and Sudan were relatively rapid assessments: they depended heavily on secondary sources, above all World Food Programme (WFP) databases on prices in markets, Food and Agriculture Organization (FAO) of the United Nations and US Department of Agriculture (USDA) data on harvests and food trade, and reports from the US Famine Early Warning Systems Network (FEWS NET), the FAO Global Information and Early Warning System (GIEWS), and humanitarian agencies. Only a few data resources from the field were collected, mainly interviews with key informants.

The studies in Nigeria and Uganda had more resources and time and so were able to gather much data from the field. Each of these two studies drew on qualitative data from focus groups and interviews with key informants, and quantitative data from a survey of households. Qualitative data were analysed to establish processes and causal paths; quantitative survey data were used to confirm the incidence and magnitude of processes observed qualitatively (see Table 1).

**TABLE 1:** Data sources.

Study area	Qualitative data	Quantitative data
Mali	Interviews with key informants in two waves: second half of 2022; and in late 2024. Reports from FEWS NET, GIEWS, and humanitarian agencies.	Food price data from WFP VAM (Vulnerability Analysis and Mapping) monthly records. FAO and USDA records of crop harvests and trade in cereals.
Sudan	Interviews with key informants in second half of 2022. Reports from FEWS NET, GIEWS, and humanitarian agencies.	Food price data from WFP VAM (Vulnerability Analysis and Mapping) monthly records. FAO and USDA records of crop harvests and trade in cereals.
Northeast Nigeria: Adamawa, Bauchi, and Gombe States	Twelve focus groups (six all‐female and six all‐male). Thirty key informant interviews. Period: April–May 2024.	Food price data from WFP VAM monthly records. Survey of 541 households.
Northern Uganda: Acholi and Lango sub‐regions	Twenty‐four focus groups. Forty‐two key informant interviews. Period: April–May 2024.	Food price data from WFP VAM monthly records. Survey of 1,670 households.

**Source:** authors.

Spatial differences are somewhat limited for Nigeria and Uganda, because the areas covered are both relatively small and geographically homogenous. For Mali and Sudan, differences across the countries are considerable, with marked contrasts between areas in conflict and more peaceful areas, and between pastoral zones (semi‐arid and arid lands) and arable zones (more humid lands). With more time and resources these differences could be analysed in detail: here, we admit that most of the information we have for Mali and Sudan concerns places with less conflict: the south and parts of central Mali and the centre and east of Sudan. The price rises that are the subject of inquiry tend to be higher in conflict zones, and their impact on populations in those areas more acute.

## 3 FOOD PRICE INCREASES AND CAUSES

**FIGURE 3:**
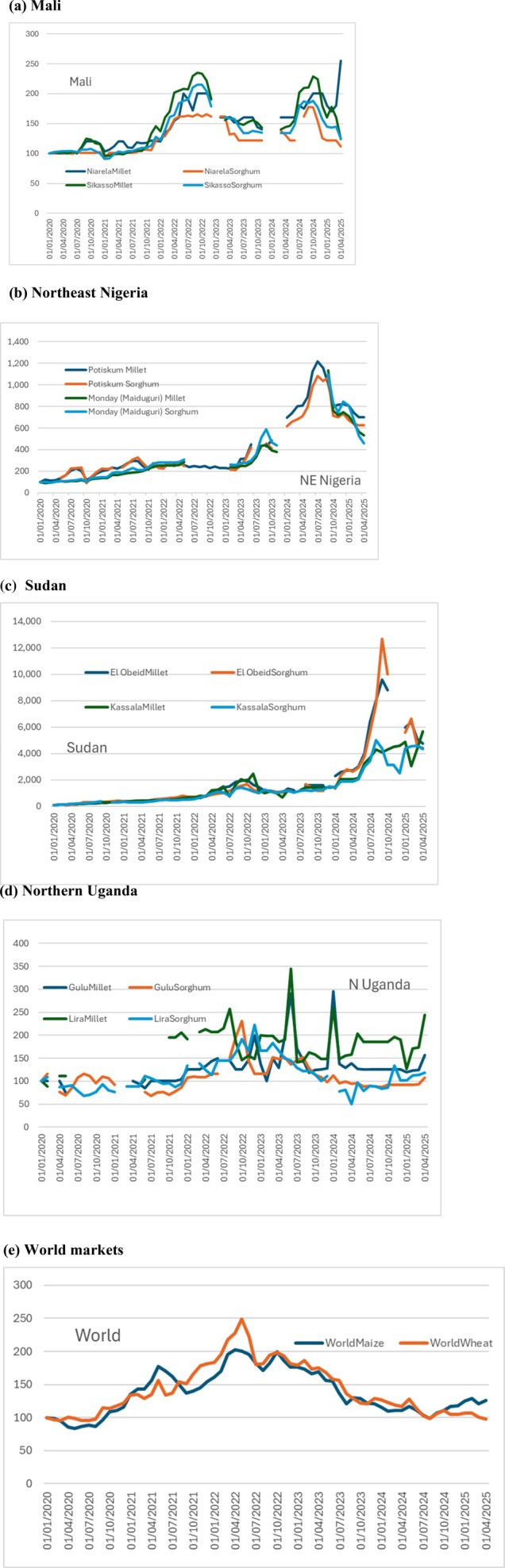
Staple food price rises from January 2020–April 2025. Prices expressed as indices, 100 = the price in January–March 2020. **(a) Mali. Note:** Niarela is a principal market in Bamako. **(b) Northeast Nigeria. Note:** Potiskum, Yobe State, and Monday, Maiduguri, Borno State, neighbour the three states studied: they were chosen because they had price records over the necessary period—the records for markets within Adamawa, Bauchi, and Gombe States were incomplete. **(c) Sudan. Note:** El Obeid is a major market in Kordofan, which has been much affected by conflict; Kassala has been impacted far less. **(d) Northern Uganda. Note:** Gulu and Lira are principal markets in Acholi and Lango, respectively. **(e) World markets. Sources:** authors, based on country price data from WFP VAM^10^; international prices from IMF Primary Commodity Prices^11^.

Movements in the prices of staple cereals in selected markets in the four countries, plus the world market prices for maize and wheat, have only one thing in common: they all increased from January 2020 (see Figure 3). After that, the differences between the five price series become striking, both in the patterns seen—above all the timing of peaks—and the magnitude of price increases.

The world prices of the two most traded grains, maize and wheat, rose from the first quarter of 2020 to peak around May 2022, when they were 2.1 times (maize) and 2.4 times (wheat) their January–March 2020 levels. Subsequently both prices fell, so that by February–April 2025 they were only 25 per cent (maize) and 4 per cent (wheat) above their levels in early 2020.

In comparison to these world market patterns, the four sets of domestic markets can be summarised as follows (see Table 2):

- **Mali**: prices doubled to a later peak, in late 2022; they have remained high through to early 2025, albeit with considerable fluctuations.
- **Northeast Nigeria**: prices peaked far later, in mid‐2024, resulting in them being more than 10 times higher than in early 2020. Those prices moderated thereafter, but by early 2025, they were still 5.7–6.4 times higher than in early 2020.
- **Sudan**: price increases were quite extraordinary, peaking in August–October 2024 at 38 to 101 times higher than in early 2020. In early 2025, they were still 37–55 times higher than in early 2020.
- **Northern Uganda**: prices rose to a first peak in October–December 2023, increasing by 0.9–1.6 times in contrast to early 2020. By early 2025, they were 0.9–2.1 times greater than in early 2020.

**TABLE 2:** Food price increases and causes.

Study	Food price changes seen since January–March 2020	Causes of price changes
Mali	To peak, November–December 2022, 1.6–2.3 times higher. To February–April 2025, 1.2–2.0 times higher.	Since 2019, production and supply of cereals have stagnated, with a notably poor harvest in 2021—owing to conflict, unaffordable and unavailable fertiliser, low rainfall, and localised floods. Common perception: traders inflate prices, but hard to test this proposition.
Northeast Nigeria	To peak, June–August 2024, 10.3–10.5 times higher. To February–April 2025, 5.7–6.4 times higher.	June 2023: the Central Bank allowed the naira to fall by 36 per cent. May 2023: fuel subsidies removed; price of fuel doubled. Inflation took off in 2023, rising by 19 per cent a year. Conflict in northeast displaced farmers. Farming hit by irregular rainfall. Prices of agricultural inputs rose three‐fold.
Sudan	To peak, August–October 2024, 36–101 times higher. To February–April 2025, 37–55 times higher.	Poor harvests in 7 of the last 10 years: available cereals per person declined. Conflict since early 2023 displaced farmers; herders blocked from usual migratory routes; trade impeded in relation to food and farm inputs; and food warehouses and processing plants damaged. Economic depression and hyperinflation made fertiliser and other inputs unaffordable. Hyperinflation (annual rates of 100–300 per cent) since 2019 helped to push cereal prices up by huge amounts.
Northern Uganda	To (first) peak, October–December 2023, 0.9–1.6 times higher. To February–April 2025, 0.9–2.1 times higher.	Droughts in 2022 and 2023. Increased food demand due to the arrival of refugees.

**Source:** authors, based on price changes computed from WFP VAM data.

Two things stand out in the record of higher prices for staples. First, the experiences of the four countries have been diverse, with more difference than commonality, reflecting as we will show, different drivers. Second, in almost all cases, consumers have faced alarming increases in the prices of staples, often far more than doubling, within a short period of time. For those on low incomes, where paying for food requires a substantial part of the household budget, the consequences have been serious.

The causes of the price rises were inferred from likely causes, considering domestic harvests, inflation, and the costs of any cereal imports, plus any other factors recorded in reports by FEWS NET, GIEWS, and other published sources (see Table 2). The degree of attribution to different factors is imprecise—it would require very detailed modelling of markets to be more specific.

Three drivers of price increases stand out. One is the supply of staples: when supply dwindles against relatively stable demand—but when increasing owing to population growth, prices rise to ration supply. This can be seen seasonally, with prices increasing from just after harvest to peak right before the next harvest, after which they fall back; trends can also be seen, though, over the medium and longer terms. In all four countries poor harvests played a role.

Mali is the exemplar: the production of cereals increased markedly from 2000–19, with total supply rising from two million tonnes (Mt) to 10 Mt, far ahead of population growth, with almost all additional supply coming from domestic farms.^12^ After 2019, however, production and supply stagnated, with a notably poor harvest in 2021. Behind the disappointing harvests lie variations in rainfall and localised floods, a large increase in the price of fertiliser stemming from the rise in international prices of urea and other fertilisers,^13^ and conflict as farmers were displaced or unable to reach their fields safely.

The supply of cereals in Sudan was severely affected by the conflict that broke out in April 2023. This exacerbated a 10‐year trend of little or no growth in domestic output: the availability of cereals, including imports of wheat,^14^ fell from 200 kilograms per person in 2016 to less than 100 kilograms per person in 2024—a very large reduction indeed.

Disappointing harvests were also recorded in northeast Nigeria owing to irregular rains, and in northern Uganda due to droughts in 2022 and 2023.

The second key driver was seen in Nigeria and Sudan: (hyper)inflation. In 2023, the Central Bank of Nigeria let the naira fall by 36 per cent, while the removal of fuel subsidies led to a doubling of the price of fuel. Although the official rate of inflation was recorded at 19 per cent a year in 2023, the prices of many everyday items, including food and fuel, rose by far more. In Sudan, high inflation could be traced back to imbalances in the balance of trade and the gap between public revenue and expenditure; imbalances managed by the control of foreign exchange and by expanding the money supply beyond the growth of the economy. These problems date back to 2012 and the loss of the south of the country and its oil revenues; however, the imbalances became more serious after 2019, and even more pressing after April 2023 and the outbreak of open conflict.

In Sudan, rising cereal prices may stem first and foremost from domestic supply lagging behind population increases. Yet, hyperinflation from 2019 explains the extraordinary size of the price rises.

The third main driver of price increases contributes to the previous factors as well as being independently important, above all in Mali and Sudan: conflict. Conflict can reduce harvests directly as insecurity leads farmers to abandon fields, crops and livestock may be destroyed or stolen, and irrigation systems are damaged; less directly, people flee their farms to avoid active fighting, trading is disrupted (because, for instance, roads become too dangerous to use, with traders being attacked and robbed), and farmers fear to invest or innovate (Özerdem and Roberts, 2012).

Just how significant the losses of agricultural output to conflict are varies greatly by the nature and intensity of conflict, which in turn can fluctuate a lot within a country: some parts of Mali (the north and increasingly the centre) and Sudan (Darfur and Kordofan) have been badly affected whereas other areas have been impacted only slightly. What perhaps is surprising is the extent to which local economies and trading still function even when conflict is active (Masset et al., 2019).^15^

Other factors reported include the widespread public perception in Mali that traders have been able use market power to hike prices. It is hard to test this contention, although if the traders can manipulate the market, then why does the record of cereal prices in Mali (see Figure 3a) show large falls as well as increases?^16^

In one case, a substantial rise in demand helped to push up prices. In northern Uganda, an influx of refugees, mainly from the Democratic Republic of the Congo and South Sudan, increased demand for cereals on local markets when harvests were faltering.

Did the higher commodity prices on world markets, as seen in the price cycle that peaked in 2022 (see Figure 1), drive these increases in domestic prices? To some extent, they must have: higher world prices for oil tend to push up the domestic prices of fuel (except in Nigeria before 2023 when the government subsidised fuel), raising the cost of transport and augmenting the prices of transported goods. The spike in the prices of fertiliser (see Figure 1) would have fed through to domestic prices in all four countries, with farmers likely applying less fertiliser with yield losses the consequence. That said, though, many of the staple crops in these four cases, above all millet and sorghum, are typically grown with very little, or no, application of manufactured fertiliser.

The effect of the spike in the prices of cereals on world markets would have been limited, owing to some of the main staples—above all millet and sorghum—not being traded, and because most markets studied lie far from the sea. Hence, a large difference applies between world and local market prices owing to transport costs.

## 4 CONSEQUENCES FOR VULNERABLE PEOPLE

*It is very difficult to cope with the rising food prices and that is a very bad experience. It is not good enough. We use ashes to wash our clothes and cow dungs for cooking. I have trouble and nagging in the home just because you ask for something to buy little food for the children. The high prices have ruined our relationship as families. It is very difficult to cope with the rising of prices and that a very bad experience for us not good enough* (Bauchi, female, married, primary education, petty trader, 30 years, interviewed in April 2024).

Confronting much higher prices for staple foods, consumers on low incomes were faced with unenviable choices: they would have to reduce food consumption, cut other spending, raise income through extra work, sell off assets (and draw down any savings), take out loans, or call on gifts and transfers. All of these ways of coping, and more, were seen in the studies reviewed (see Table 3). For Mali and Sudan, we only have qualitative information; for Nigeria and Uganda, household surveys allowed estimation of incidence and degree of coping.

**TABLE 3:** Coping with higher food prices.

Coping method	Mali	Northeast Nigeria	Sudan	Northern Uganda
Changed consumption: food	People on low incomes switched from more expensive to cheaper foods, and reduced food consumption entirely. Conflict prevented foraging for food in the bush. Reports of increased malnutrition.	More than 90 per cent of households reported reduced access to food. 91 per cent reduced meal frequency. 42 per cent switched to cheaper foods of lower quality. Breastfeeding mothers reported difficulties owing to poor nutrition.	People bought cheaper and often less preferred food, limited portion sizes, or reduced the number of daily meals. In areas of active conflict there were reports of consuming wild foods, although insecurity made gathering risky. Widespread food insecurity and malnutrition: famine declared in parts of Darfur by 2024.	More than 80 per cent of households bought cheaper food, changed diets, or cut the size of meals. 34 per cent visited health centres as a consequence of eating one meal a day; 20 per cent took a hungry child to health centre.
Changed consumption: health	Spending on medical care fell.	Between 48 and 85 per cent of households (depending on gender and state) could no longer afford medicines. In Gombe, most respondents said they could no longer afford to visit health centres.	Health services were reported to be barely functioning by 2024.	Reports of increased visits to health centres on account of malnutrition and its consequences.
Changed consumption: education	Some parents had taken their children out of school to save on fees and expenses.	Some parents withdrew children from school. They were either unable to afford schooling costs or needed the child to work. Instances of child labour and begging increased.	Not reported.	40 per cent moved a child to a cheaper school; 37 per cent withdrew a child from school; and 21 per cent married off a teenage girl.
Search for extra income: jobs and work	Mentioned most frequently: young men to informal gold mining and young women to towns. Seen as negative.	32 per cent sought additional income‐generating activities. 63 per cent of males and 51 per cent of females migrated to find work.	Where available, people took up informal jobs: but a depressed economy meant that there was no work or wages were very low. Emigration by young men to find work in other countries.	57 per cent took up extra work, including: petty trade (27 per cent); casual labour in agriculture (9 per cent); vegetable gardening (7 per cent); and livestock keeping (7 per cent).
Search for extra income: remittances	Implicit in frequent reports of migration.	Not reported.	Remittance flows intermittent owing to disrupted telecommunications and banking.	8 per cent of households reported receiving remittances from family members.
Search for extra income: asset sales	(Not mentioned, but national survey shows this to be common.)	80 per cent of males and 72 per cent of females sold assets. Most often these were: sheep or goats (43 per cent); furniture (35 per cent); clothing (33 per cent); land (28 per cent); and cooking utensils (18 per cent).	Asset sales reported; few details available. Large‐scale farmers sold off stored crops. Herders sold off livestock.	57 per cent sold assets, including some who sold seed.
Search for extra income: credit	(Not mentioned, but national survey shows this to be common.)	34 per cent of households borrowed money.	Not reported.	71 per cent took out a loan, overwhelmingly from VSLAs (34 per cent) and family or friends (25 per cent). Less than 4 per cent took out credit from microfinance agencies, banks, landlords, or money lenders.
Social distress: violence, stress, and sex work	Several mentions of social ills: resort to crime, sex work, begging, and to joining insurgent militias.	Tension between spouses increased. Men left home to avoid being asked for money for food. Breakdowns in communication and depression and anxiety.	Not reported.	55 per cent of households reported domestic violence. 46 per cent observed commercial sex work in their community; 9 per cent admitted that they resorted to such activity. 72 per cent reported the theft of livestock.

**Source:** authors.

### 4.1 Reduced consumption

#### 4.1.1 Food and nutrition

Most people reported changes in food consumption. Typically, people on low incomes switched their spending on food from any expensive items, including animal foods and ‘luxury’ staples like rice and bread, to cheaper options such as millet, sorghum, and cassava (42 per cent of households in Nigeria). Even that was not enough for some: reduced portion sizes and fewer meals were commonly reported (91 per cent of households in Nigeria and 80 per cent of households in Uganda). Two study participants commented:

*Now I cannot eat food anytime I feel like eating. When prices of food commodities were cheap, I eat food at least three times in a day. But now, I eat at the most twice a day. Even those two times are not to satisfaction* (Gombe, male, married, Islamic/Arabic education, farmer, 40 years, interviewed in April 2024).

*Since 2022, the impact has been the reduction in the number of meals consumed per day: from three meals to two meals or even one (meal per day)* (Bamako, development project field officer, interviewed in December 2024).

In Mali, people found that foraging for wild foods in the bush was no longer safe in conflict zones. One said:

*Unfortunately, the other people did not dare to go into the bush in search of millet because of the ‘terrorists’* (Bandiagara, head of a women's cooperative, interviewed in December 2024).

In Sudan, vulnerable households skipped and reduced meals, and switched from more costly to cheaper items, substituting, for example, pigeon peas for meat or millet for rice. In the country's conflict zones, especially in Darfur, by 2023, people without work, people who had lost livestock, and people who were internally displaced were resorting to wild foods—fruits, vegetables, seeds, grasses, and leaves. There was even mention of some resorting to bark, twigs, peanut shells, and dirt. Again, conflict meant that foraging was not safe.

Poorer nutrition was often reported. In Uganda, 34 per cent of households visited health centres because of eating one meal a day; 20 per cent of households took a hungry child to such a facility. Households resorting to cheaper food were twice as likely to report child malnutrition as compared to households on a regular diet. Humanitarian agencies assessed much of Sudan as being in food crisis (IPC Phase 3) by 2024, areas of conflict as being in food emergency (IPC Phase 4), and some parts of Darfur as in famine (IPC Phase 5)—an assessment not reached lightly, but justified by evidence of widespread severe and acute child malnutrition.

#### 4.1.2 Health and education

*A major part of our income is tied towards buying food, we cannot even afford to buy medication when our children fall sick now. Some of our children have to be withdrawn from their schools because we cannot afford to pay their school fees* (Gombe, male, married, Islamic/Arabic education, businessperson/farmer, internally displaced, 37 years, interviewed in April 2024).

With food budgets under strain, households reported spending less on medicines and healthcare. In northeast Nigeria, 48–85 per cent of households could no longer afford medicines: in Gombe State, most households could no longer afford to visit health centres.

In Sudan, conflict meant that many health services were barely operating by 2024. In Uganda, some households reported visiting health centres more often to seek a remedy for malnutrition and its consequences. One study participant stated:

*Our children are becoming malnourished because we can't afford proper food. The hospitals are full of weak children, and we worry about their future health* (Pader, female, interviewed in April 2024).

A common result of higher food prices was *economising on education* and its costs, moving children to schools with lower fees (40 per cent of households in Uganda) or withdrawing children from school entirely (37 per cent of households in Uganda). In Nigeria, taking children out of school was also motivated by the need to put their children to work—or to go begging. One study participant underlined:

*I am supposed to pay before school resumes; I now struggle to pay half at the end of the term. This has made the school always send my children back home several times. You see, this has affected both the children and my reputation as well* (Gombe, male, married, master's, businessperson, 53 years, interviewed in April 2024).

In Uganda, 21 per cent of households had taken adolescent girls out of school and married them off as teenage brides. This was to save on household costs, to avoid falling into debt, to attract a bride price, and to rescue girls from commercial sex work.

### 4.2 Generating extra income

#### 4.2.1 Searching for extra work

In all four countries, families looked to generate additional income, from formal to informal work. In Mali, the most frequent reports were of the young leaving villages to seek work: young men to informal gold mines and young women to towns to seek domestic work. For instance:

*[This] has led to the mass exodus of young people to gold panning sites* (Bankass, secondary school teacher, interviewed in December 2024).

*[This has led to the] early departure of girls from rural areas to urban centres* (Ségou, humanitarian project manager, interviewed in December 2024).

Those interviewed in Mali who commented on such migration considered the developments to be an evil.

In Nigeria, 32 per cent of households reported taking up activities to generate an income, but more than one‐half of adults, both male and female, migrated (largely within Nigeria) to find extra work.

In Sudan, people looked for informal jobs, but with an economy depressed by the effects of conflict, these were scarce or came with very low wages. Opportunities included working as security guards or in informal gold mining. Some young men emigrated to find work in Egypt, Saudi Arabia, and the Gulf states, or even further afield. All of these options, though, implied the risk of moving across fighting zones.

In Uganda, slightly more than half of the households took on extra work, mainly involving petty trade, casual agricultural work, vegetable gardening, or keeping livestock—although some migrated looking for casual work in factories such as sugar mills. One participant said:

*I started selling vegetables at the market to survive, but it's barely enough to feed my family. We are living day by day* (Pader, female, interviewed in April 2024).

#### 4.2.2 Asset sales and remittances

*I had to sell my goats and some land to cover food expenses. Now, I have nothing left to fall back on* (Uganda, interviewed in April 2024).

Assets were commonly sold in all four countries (by 80 per cent of males and 72 per cent of females in Nigeria and by 57 per cent of households in Uganda). In Sudan, larger farmers sold off stored crops; herders sold more livestock than normal. While replaceable assets were often sold—small livestock, cooking utensils, clothing, and household items—land sales were also mentioned in Nigeria (by more than one‐quarter of households) and Uganda.

Where people had migrated, their households might then receive remittances. Remittances were not necessarily common: just 85 of 1,670 households in Uganda reported receiving them. In Sudan, conflict disrupted banking and telecommunications so that remittances were, at best, intermittent.

#### 4.2.3 Borrowing

Seeking credit was another common response in Mali and Uganda. Notably, 71 per cent of households in Uganda took out loans: 34 per cent from Village Savings and Loans Associations (VSLA) and 25 per cent from family members, but less than 4 per cent from banks, microfinance agencies, landlords, or money lenders). Borrowing took place in Nigeria and Sudan, but the extent is not known. One study participant remarked:

*I have gone into so many debts because my business is dying. I have incurred so many debts that I sometimes hide on my way to work or business* (Gombe, male, married, tertiary education, civil servant, 56 years, interviewed in April 2024).

#### 4.2.4 Shameful and illicit activities

Hard times led some to resort to petty crime, gambling, commercial sex work, and begging (often by children); in Mali, youths were reported to have joined insurgent militias. In Uganda, only 9 per cent of interviewees admitted to commercial sex work, but 46 per cent said that people in their community were so engaged, suggesting that such activity was more common than those engaged in it were prepared to admit. In the words of one interviewee:

*Children are engaging in stealing and other crimes just to get food. The girls engage in prostitution while others are begging on the street. Child labour is happening here. Many children are out of school because we cannot afford to buy learning materials for them; even the clothes we buy before we cannot buy for them now* (Adamawa, male, married, tertiary education, businessperson, 42 years, interviewed in April 2024).

### 4.3 Social trauma

Coping took its toll on those affected, especially women and girls: not only did they have to cope with limited means to feed their families and attend to sick children, but also girls were most likely to be taken out of school, sometimes to become child brides. Not surprisingly, many women and girls emphasised their personal stress and conflict within the household.

In Mali, increased conflict within households was commonly cited: indeed, some observers commented that coping with higher prices (plus conflict and a stuttering economy) was demoralising, with frustration manifesting as bad temper and petty arguments. One study participant encapsulated this:

*Youth unemployment, prostitution of young girls, alcoholism, smoking, banditry among the juvenile strata, school dropout, and prey to recruitment for terrorism* (Ségou, humanitarian project manager, interviewed in December 2024).

In Nigeria, coping put a heavy strain on women trying to manage their households. For instance:

*We women, who often oversee household budgeting and meal planning have experienced increased stress and anxiety as we navigate the challenges of stretching limited resources to feed our families* (Adamawa, female, married, primary education, petty trader, 47 years, interviewed in April 2024).

*Every little thing I say to my husband, he gets offended. He usually goes out of the house once it's almost time for cooking food to avoid being asked money for anything* (Bauchi, female, married, primary education, petty trader, internally displaced, 53 years, interviewed in April 2024).

In Uganda, 55 per cent of households reported violence at home and 17 per cent spoke of divorce and separation, although it is not clear to what extent coping increased these problems. In the words of one participant:

*My husband has become more aggressive since the prices went up. He drinks more, and there are constant arguments because there is no money for food* (Pader, female, interviewed in April 2024).

Women and girls bore the brunt of the hardship, but men suffered too. In Nigeria and Uganda, men reported feeling ashamed of their inability to provide for their families, leading some to spend as little time at home as possible. For example:

*Only God can interpret the level of anxieties and pains, including shame, I pass through always due to my helplessness to provide for the family* (Bauchi, male, married, national diploma, farmer/trader, 64 years, interviewed in April 2024).

*As the head of the house, we are expected to provide for our families; we had to get more non‐formal jobs to get money to feed our families, resulting in stress, anxiety, and health challenges. I have been a victim. Any man who cannot provide for his family sees himself as a failure. This is the root of the problem and stress* (Adamawa, male, married, tertiary education, businessperson, 42 years, interviewed in April 2024).

## 5 PUBLIC RESPONSES

Most people affected by higher food prices in the four countries under review received very little, if any assistance, from public agencies, whether central or local government, NGOs or CSOs, or an aid partner or humanitarian agency. Uganda was the exemplar: just seven per cent of households received support from public agencies, most commonly government, primarily as cash transfers—however, these were not a response to higher food prices, but rather were part of a national social protection programme, with transfers targeted at the elderly. Another 23 per cent of households reported getting help, but not from public agencies: they received occasional assistance from family members who sent remittances or from ‘good Samaritans’ in the local community.

A larger proportion of the population in northeast Nigeria—35 per cent of male and 37 per cent of female respondents—received public assistance. Most had been given food or a cash transfer; less commonly they were provided with subsidised farm inputs and training. Most recipients, though, thought that what they received was only slightly adequate or inadequate.

In Mali, the government and local NGOs tried to assist people hit by higher prices, distributing foods at little or no cost. But the scale of these efforts was limited as compared to need.^17^ Mali also has been receiving international humanitarian aid in response to several crises that have affected the population, including disasters triggered by drought and floods, as well as conflict and the associated displacement of the domestic population and refugees from neighbouring countries. In 2024, the United Nations Office for the Coordination of Humanitarian Affairs (UN OCHA), as coordinator of international relief, assessed that of more than 22 million Malians, 7.1 million persons needed relief, of which 4.1 million were targeted but only 2.1 million were reached. Relief needs were budgeted at USD 702 million, but by June 2025, only 33 per cent of that sum, USD 235 million, had been funded. The amount equates to an average of USD 171 per person targeted; but the funding received would be worth only USD 57 per person.^18^

Sudan has seen some of the largest humanitarian relief operations in the world in the 2020s. The scale of this effort is impressive, although insufficient to meet assessed needs. As of early July 2025, the United Nations (UN)‐coordinated *Sudan Humanitarian Needs and Response Plan 2025* calculated that 30.4 million people required humanitarian assistance, with 20.9 million targeted by the UN and its partners. The plan appealed for USD 4.2 billion, with USD 3 billion required urgently. Funding commitments by 9 July 2025 had reached USD 916.9 million, 22 per cent of the appeal level. Between January and May 2025, only 12.4 million people (60 per cent of the target of 20.9 million and 41 per cent of the 30.4 million requiring assistance) had received humanitarian assistance, including 8.7 million who had received food assistance and 2.7 million who had received health support (UN OCHA, 2025).^19^

In all four cases, owing to a lack of public assistance, those most affected by higher prices—and in Mali and Sudan, by conflict and disasters resulting from drought and floods—have had to fall back on the resources of the household, family, and local community. Mutual aid at the community level has sometimes been significant: in Sudan, gifts (sometimes as part of *zakat*) and food sharing were commonly seen (Birch, Carter, and Satti, 2024; FEWS NET, 2024).

Some innovative responses were reported in northeast Nigeria, where communities had formed cooperatives to buy items in bulk. In Sudan, Emergency Response Rooms (ERRs) in major cities represent a more formalised type of mutual aid, providing, among other things, community kitchens and medical services (FEWS NET, 2024).

## 6 DISCUSSION AND CONCLUSION

### 6.1 Summarising the key points

From early 2020 in all four countries, the prices of staple foodstuffs rose by large amounts over the next two years, if not longer. Price rises in northeast Nigeria and Sudan were extraordinary. In very large part, these can be attributed to domestic and local factors, notably failed harvests owing to natural hazards; in Mali and Sudan, they can be ascribed to conflict. World prices have played only a minor role.

People on low incomes have been hard hit by the rise in price of staple foods: they have not only been forced to economise on food but also have had to cut spending on health and education. Many people so impacted have tried to introduce coping mechanisms such as finding extra work, selling off assets, and borrowing money. In the process, people have also suffered mental and physical distress.

Public support, from the government or NGOs or donors, has been scant: most people have had to manage using the resources of the family, neighbours, and local communities.

### 6.2 Discussion: policy implications

**Can governments do more to stabilise the prices of staple foods?** One solution, often mentioned, is that the government might keep stocks of cereals to be released when harvests fail or supplies run short for whatever reason. It is, however, costly to create and manage a reserve large enough to stabilise prices (Poulton et al., 2006)^20^; and the costs of large public food reserves are simply unaffordable in most low‐income countries. An alternative to centralised food reserves is to encourage community stores, but the costs of such reserves are also considerable (Pons Cortès and Gómez, 2013).

Price rises would almost certainly be mitigated if there were more trade to link places experiencing harvest failures with those that have experienced good harvests. Progress towards easier trade across Africa is being made, particularly in the East African Community, complemented in the past few years by the creation of the Africa Continental Free Trade Area, which should help (Janssens et al., 2022).

That said, the lack of imports to Mali, Nigeria, and Sudan was surprising. In November 2024, the world prices of maize and sorghum were USD 202 and USD 242 per tonne, respectively.^21^ But in that month, the price of sorghum on domestic markets was far higher: USD 610 per tonne in Bamako, Mali; USD 980 per tonne in Kassala, Sudan; and a staggering USD 1,224 per tonne in Maiduguri, Nigeria.^22^ While these are all inland areas requiring costly overland transport of imports from the world market, such costs are a small share of the difference between domestic and world prices—for example, road freight from Abidjan to Bamako costs less than USD 200 per tonne. It is hard to explain the failure of traders to take advantage of these price differentials by importing: it may be owing to differences in the quality of sorghum available on world markets (most is traded as livestock feed), or it may be due to policies, taxes, and tariffs to discourage imports.

In the medium term, variations in harvests may be reduced: by research into crops resistant to drought, flooding, extreme heat, and (specific) pests and diseases; by irrigation; and by developing farming systems that conserve soil and water—matters central to the agenda of climate‐smart agriculture (Descheemaeker, Reidsma, and Giller, 2020). Much research, both formal and informal—by farmers drawing on indigenous knowledge (see, for example, Reij et al., 2020)—has been done on such technologies over the past 40 or more years. While more research will yield dividends, the greater challenge may well be to adapt existing knowledge to local circumstances and encourage the adoption of the technologies developed.

Last, but far from least, bringing conflict to an end would do much to allow farming and other local activity to recover, above all in Mali and Sudan.

**Can public agencies do more to alleviate hardship among those most affected by higher food prices?** Governments, NGOs, and international donors have limited funds to support people hit hard by food crises. Despite much increased interest in social protection since 2000, and particularly cash transfers to vulnerable people, cover is still scant across most of Africa (Beegle, 2018).^23^ The ideal of a social protection net that substantially eases hardship for most people affected by higher food prices remains, for the time being, a pipe dream.

The lesson from these cases is that most people must try to cope using their own and localised resources. Any additional public action should look to complement and reinforce the (most successful) coping mechanisms that people utilise. Examples of effective coping actions from the four countries under review include VSLAs in northern Uganda, collective bulk buying in northeast Nigeria, and ERRs in Sudan.

**How can especially vulnerable persons be protected from the long‐term consequences of food crises?** Children may see their lives blighted by a food crisis, even if it lasts only a few years. Infants, for instance, may be malnourished to the point where their physical and mental growth is impeded. Monitoring the growth of infants in vulnerable populations, and taking remedial action, becomes especially important during food crises.^24^ School‐age children, girls especially, may be removed from school to avoid the costs of education and put to work at home or engaged in employment. Public assistance targeted at vulnerable households to allow them to pay fees and other costs of schooling will probably have major long‐term benefits.

A final short reflection concerns the nature of contemporary food crises: are they similar to those seen in the past, or are they novel in terms of their frequency, severity, or nature? The only obvious novelty in these cases is the rising incidence of natural hazards, such as droughts and floods, almost certainly stemming from global warming. Otherwise, these crises resemble those of the past. Their frequency may be rising as a consequence of climate change, but their severity may be less than has been in the past. Public agencies have more capacity—experience, budgets, and trained staff—today than previously: it should be possible, therefore, to respond more adequately to these events.

## CONTRIBUTIONS

*Research planning, data collection, and initial analysis*: Bilkisu Yayaji Ahmed, Betty Akullo, Boukary Barry, Johnson Dudu, Job Eronmhonsele, Yusuf Kiwala, Dicta Ogisi, Andrew Onokerhoraye, Jimmy Opio, Neema Patel, Hussein Sulieman, and Steve Wiggins.

*Analysis of prices and other economic data*: Steve Wiggins.

*Lead writer and corresponding author*: Steve Wiggins.

## CONFLICT OF INTEREST STATEMENT

The authors have no conflicts of interest to declare.

## Data Availability

The data that support the findings of this study are available from the corresponding author upon reasonable request.

## Endnotes

^1^
The invasion led to a flurry of blogs discussing the implications for world prices of cereals, fertiliser, and oil and the likely threat to food security in the Global South. Commentaries on overall impacts were drafted by research institutes and think‐tanks (Mitchell, Hughes and Huckstep, 2022; Gill, 2022; Glauber and Laborde, 2022; Smith, 2022), as well as by the business press (*The Economist*, 2022; The Editorial Board, 2022). Specific concerns were expressed about the impact on Egypt and Yemen, countries heavily dependent on wheat imports from Russia and Ukraine (Abay et al., 2022; Kurdi et al., 2022; Belhaj, 2022).

^2^
The previous cycle saw a spike in late 2007–08, with lesser ones recurring in 2011 and 2012. The prices of cereals, in constant terms, returned to their pre‐spike levels by 2016. The 2007–08 spike was much larger than that seen in 2022: prices rose in real terms by 139 per cent for maize, by 125 per cent for wheat, and by 209 per cent for rice (Wiggins and Keats, 2013).

^3^
The exposure of Kenya, Mali, Sudan, and Yemen to international price increases for cereals and fertiliser was appraised (Wiggins, 2022). The food systems of the Ten Plus One group of countries (14 in all, 11 of them SPARC nations) that FCDO considered to be particularly at risk of food crisis and emergency were scanned to understand their vulnerability to higher world prices (Mendez‐Parra et al., 2023).

^4^
See https://www.ifpri.org/topic/food-crises/ (last accessed on 24 November 2025).

^5^
See https://www.ipcinfo.org/ipcinfo-website/ipc-overview-and-classification-system/en/ (last accessed on 24 November 2025).

^6^
Heltberg, Hossain, and Reva (2012, p. 1) wrote, in regard to the consequences for world markets of the 2007–08 food price spike and the 2008 financial crisis: ‘Systemic shocks of the complexity and scale witnessed from 2008 through 2011 are quite unprecedented in world history’. Complexity and scale are matters for judgement: in terms of their ability to kill people, however, shocks in the twenty‐first century are mercifully low as compared to the appalling death rates seen in Africa when Europe invaded.

^7^
See https://worldpeacefoundation.org/ (last accessed on 24 November 2025).

^8^
See https://www.aehnetwork.org/data-research/african-population-database-1850-1960/ (last accessed on 24 November 2025).

^9^
See https://www.fao.org/faostat/en/#home (last accessed on 24 November 2025).

^10^
See https://dataviz.vam.wfp.org/ (last accessed on 25 November 2025).

^11^
See https://www.imf.org/en/Research/commodity-prices (last accessed on 25 November 2025).

^12^
Imports, mainly of rice and wheat, also rose over the period, but even by 2024, imports made up just 7.5 per cent of total supply.

^13^
This made little difference, however, to the supply of the cheapest cereals, millet and sorghum, where little manufactured fertiliser has ever been applied.

^14^
Wheat imports into Sudan made up between 18 and 34 per cent of total supply from 2016–24, with larger shares in years when domestic harvests were low.

^15^
Also notable here were personal communications from humanitarian workers in Sudan.

^16^
The cereals market analyses of FEWS NET and GIEWS do not mention the manipulation of markets.

^17^
The Government of Mali also tried to regulate food markets and trading, setting maximum prices, subsidising imports of rice and sugar, and banning cereal exports. Informants commented that price regulation had been ineffective owing to a lack of effective enforcement.

^18^
For more information, see https://humanitarianaction.info/plan/1194 (last accessed on 18 December 2025).

^19^
The same source (UN OCHA, 2025) also reported that 24.6 million people were facing food insecurity of IPC Phase 3 or above, including 637,000 persons who were confronting catastrophic food insecurity (IPC Phase 5) by June 2025. Additionally, 3.7 million children under five years of age and pregnant and breastfeeding women needed treatment for acute malnutrition in 2025.

^20^
Public food stores were much discussed in Southern Africa in 2002 when six countries of the region experienced harvest failures with subsequent food crises (Tschirley et al., 2005; Tschirley and Jayne, 2010).

^21^
See https://www.imf.org/en/Research/commodity-prices (last accessed on 25 November 2025).

^22^
Official exchange rates used for Mali and Nigeria; the parallel rate was used for Sudan.

^23^
Exceptions are few—Ethiopia, Ghana, Kenya, Senegal, and Tanzania: the considerable literature on some of these programmes, above all Ethiopia's Productive Safety Net Programme, obscures the low coverage across much of the rest of Africa.

^24^
The effects of malnutrition may be offset by advances in public health: to reduce disease that exacerbates the reduced intake of food, and indeed has been the main cause of mortality among children aged under five years. Across much of rural Africa, infant mortality, under‐five child mortality, and stunting rates have fallen by large amounts since the early 1990s. Investment in basic healthcare has paid off: such efforts should be sustained and intensified.
